# Polymorphisms of *CUL5* Are Associated with CD4^+^ T Cell Loss in HIV-1 Infected Individuals

**DOI:** 10.1371/journal.pgen.0030019

**Published:** 2007-01-26

**Authors:** Ping An, Priya Duggal, Li Hua Wang, Stephen J O'Brien, Sharyne Donfield, James J Goedert, John Phair, Susan Buchbinder, Gregory D Kirk, Cheryl A Winkler

**Affiliations:** 1 Laboratory of Genomic Diversity, SAIC-Frederick, Incorporated, National Cancer Institute, Frederick, Maryland, United States of America; 2 Inherited Disease Research Branch, National Human Genome Research Institute, Baltimore, Maryland, United States of America; 3 Basic Research Program, SAIC-Frederick, Incorporated, National Cancer Institute, Frederick, Maryland, United States of America; 4 Laboratory of Genomic Diversity, National Cancer Institute, Frederick, Maryland, United States of America; 5 Rho Incorporated, Chapel Hill, North Carolina, United States of America; 6 National Cancer Institute, Bethesda, Maryland, United States of America; 7 Northwestern University, Chicago, Illinois, United States of America; 8 San Francisco Department of Public Health, San Francisco, California, United States of America; 9 Bloomberg School of Public Health, Johns Hopkins University, Baltimore, Maryland, United States of America; The Jackson Laboratory, United States of America

## Abstract

Human apolipoprotein B mRNA editing enzyme, catalytic polypeptide-like 3 (Apobec3) antiretroviral factors cause hypermutation of proviral DNA leading to degradation or replication-incompetent HIV-1. However, HIV-1 viral infectivity factor (Vif) suppresses Apobec3 activity through the Cullin 5-Elongin B-Elongin C E3 ubiquitin ligase complex. We examined the effect of genetic polymorphisms in the *CUL5* gene (encoding Cullin 5 protein) on AIDS disease progression in five HIV-1 longitudinal cohorts. A total of 12 single nucleotide polymorphisms (SNPs) spanning 93 kb in the *CUL5* locus were genotyped and their haplotypes inferred. A phylogenetic network analysis revealed that *CUL5* haplotypes were grouped into two clusters of evolutionarily related haplotypes. Cox survival analysis and mixed effects models were used to assess time to AIDS outcomes and CD4^+^ T cell trajectories, respectively. Relative to cluster I haplotypes, the collective cluster II haplotypes were associated with more rapid CD4^+^ T cell loss (relative hazards [RH] = 1.47 and *p =* 0.009), in a dose-dependent fashion. This effect was mainly attributable to a single cluster II haplotype (Hap10) (RH = 2.49 and *p =* 0.00001), possibly due to differential nuclear protein–binding efficiencies of a Hap10-specifying SNP as indicated by a gel shift assay. Consistent effects were observed for CD4^+^ T cell counts and HIV-1 viral load trajectories over time. The findings of both functional and genetic epidemiologic consequences of *CUL5* polymorphism on CD4^+^ T cell and HIV-1 levels point to a role for Cullin 5 in HIV-1 pathogenesis and suggest interference with the Vif-Cullin 5 pathway as a possible anti-HIV-1 therapeutic strategy.

## Author Summary

Human apolipoprotein B mRNA editing enzyme, catalytic polypeptide-like 3 G (Apobec3G) is an innate antiviral protein that inhibits HIV type 1 (HIV-1) replication by causing deleterious mutations in the HIV-1 genome. Unfortunately, HIV-1 has a strategy to defeat the antiviral activity of Apobec3G. The HIV-1 viral infectivity factor (Vif) binds to Apobec3G leading to the degradation of Apobec3G through a complex containing Cullin 5 and the proteins Elongin B and Elongin C. Since Cullin 5 directly interacts with Vif and is critical to the Apobec3G degradation pathway, the authors asked if genetic variation of *CUL5* could tip the balance between HIV-1 and Apobec3G and modify the course of HIV-1 infection. They showed that genetic variation in the *CUL5* gene encoding Cullin 5 affected the rate of CD4^+^ T cell loss in patients infected with HIV-1. *CUL5* haplotypes formed two clusters of evolutionarily related haplotypes with opposing effects—cluster I delayed and the cluster II accelerated CD4^+^ T cell loss. The effect was mainly attributable to a single haplotype or its tagging-SNP, which demonstrated differential binding of transcription factors. This finding highlights the epidemiologic importance of the HIV-1 and Cullin 5 interaction and suggests that the factors in the HIV-1 Vif-Apobec3G degradation pathway may be targets for antiviral drugs.

## Introduction

Members of the apolipoprotein B mRNA editing enzyme, catalytic polypeptide-like 3 (Apobec3) family of cytidine deaminases are innate cellular anti-HIV-1 factors [1,2]. In the absence of HIV-1 viral infectivity factor (Vif), both Apobec3G and Apobec3F are packaged into HIV-1 virions and during reverse transcription in the newly infected cell deaminate dC to dU in the nascent minus-strand DNA. This deamination results in either the degradation of the cDNA through a cellular uracil-DNA-glycosidase degradation pathway or pervasive G to A hypermutation in the plus-strand proviral cDNA [3–7]. However, the antiretroviral activities of Apobec3G and Apobec3F are suppressed by HIV-1 Vif, effectively preventing incorporation of Apobec3G or Apobec3F into virions, primarily by inducing Apobec3G degradation by proteasomes [8–11], and perhaps by additional mechanisms [5,12,13]. HIV-1 Vif interacts with the cellular proteins Cullin 5, Elongin B , Elongin C, and Rbx1 to form an E3 ubiquitin ligase complex that induces polyubiquitination and proteasomal degradation [7]. When the Cullin 5 complex is inhibited by mutating Cullin 5 or is down-regulated by RNA interference, Vif-induced polyubiquitination and degradation of Apobec3G is blocked [7,14]. This suggests that the ability of HIV-1 Vif to suppress the antiviral activity of the two Apobec3 proteins specifically depends on Cullin 5-Elongin B-Elongin C function [7,14]. Most recently, the Vif-Cullin 5 binding domain has been mapped to a highly conserved HCCH motif within the HIV-1 Vif zinc-binding domain [15,16]. The region in Cullin 5 that mediates Vif interaction has been mapped to the loop region between helices 6 and 7 (amino acids 120–138) [16]. In an independent report, the Vif interaction region was mapped to the first cullin repeat (amino acids 1–158) of Cullin 5 [17].

We recently reported that a nonsynonymous single nucleotide polymorphism (SNP) in the *APOBEC3G* gene may be associated with altered AIDS progression [18]. Since Cullin 5 is a critical host factor in the Vif-mediated degradation pathway of anti-HIV-1 proteins Apobec3G and Apobec3F, we investigated the effects of genetic variation in *CUL5* on the natural history of HIV-1 progression.

## Results

### Description of *CUL5* Variants

**Figure 1:**
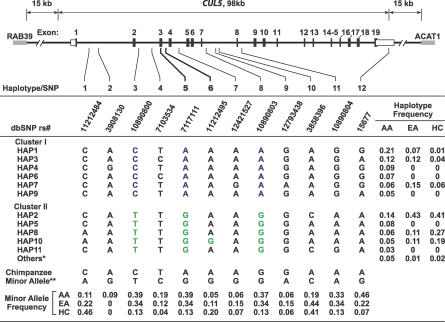
Gene Map, SNPs, and Haplotypes in the Human *CUL5* Gene Coding exons are marked by black blocks, and 5′ and 3′ UTR by white blocks. Nucleotide changes and frequencies of SNPs and haplotypes (Hap) in AA, EA, and HC are presented. ctSNPs are shown in color.

The *CUL5* gene is approximately 100 kb in length and consists of 19 exons (Figure 1). At the start of this project, about 40 SNPs for the *CUL5* gene on human Chromosome 11q22 had been deposited in the National Center for Biotechnology Information database SNP (NCBI dbSNP) (http://www.ncbi.nlm.nih.gov/SNP/). No nonsynonymous nucleotide replacements have been reported to date. Through resequencing of 188 DNA samples, we did not discover any additional SNPs in exons 15–19 in the C terminus of the *CUL*5 gene or in the putative promoter region. By considering SNP location, spacing, and allele frequency, 12 SNPs were selected for genotyping in the AIDS cohorts (Figure 1 and Table S1). Of these 12 SNPs, one SNP (SNP5, rs7117111) is within the coding region, one (SNP12) is in the 3′ UTR, and the remaining SNPs are in introns. SNP5 is a synonymous transition (CAA > CAG) at the third position of codon 75 encoding glutamine (Q). Each of the 12 SNPs was in Hardy-Weinberg equilibrium (*p* > 0.05) in African Americans (AA) and European Americans (EA).

### *CUL5* SNPs Exhibit Strong Linkage Disequilibrium

**Figure 2:**
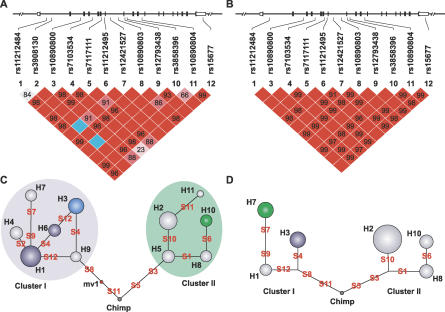
LD of *CUL5* SNPs in AA and EA LD of *CUL5* SNPs is shown in AA (A) and EA (B). Pairwise D′ plots were generated using Haploview with its standard color scheme. Dark-red squares indicate high D′ values, light-blue squares indicate high D′ values with low LOD scores, and light-red and white squares indicate low D′ values. D′ values were indicated for those not equal to 1.0. A single LD block was defined for both AA and EA under the default confidence interval criteria. A reduced-medium network for the genealogical relationship of *CUL5* haplotypes is shown in AA (C) and EA (D). The network was inferred in terms of mutational distance, on the basis of 12 *CUL5* SNPs and one chimpanzee (Chimp) sequence. Median vector (mv1), the consensus sequences inferred by parsimony criteria, represents possible unsampled sequences or extinct ancestral sequences. Haplotypes (H1–H11) are represented by circles, whose area reflects the number of alleles observed in each population. The solid branches between haplotypes represent mutational events or SNPs (S1–S12). The circles in green show haplotypes with detrimental effect and those in blue show protective effect on AIDS progression in the Cox model analysis; the protective effect of H3 in light blue was of less certainty (see Results). The haplotypes were separated into two clusters, cluster I and II, carrying ctSNP5 A or G, respectively. Cluster I and II in AA are shaded in blue and green, respectively. SNP2 is omitted in (B) and (D) as it was absent in EA.

The 12 SNPs span 93 kb of the *CUL5* gene (Figure 2A and 2B). The extent of linkage disequilibrium (LD) was assessed by calculating all pairwise D′ values among the *CUL5* SNPs, separately for AA and EA (Figure 2A and 2B). Strong LD was observed among all SNPs (D′ range 0.92–1.0) in EA and almost all SNPs in AA, with the majority of marker pairs showing D′ values between 0.95 and 1.0. In both AA and EA, as well as in a Han Chinese (HC) population, SNPs 3, 5, and 8 and SNPs 7 and 9 were in perfect LD (D′ = 1 and *r* ^2^ = 1). Thus each SNP carries the same information content as its proxies. We therefore used SNP5 as a proxy for SNPs 3 and 8 and SNP7 as a proxy for SNP9. SNP5 has a diverse allele distribution in different population groups: the A allele frequency is 0.61, 0.34, and 0.13 in AA, EA, and HC, respectively. Only a single LD block formed by all 12 SNPs was identified for both population groups as defined by confidence intervals of pairwise D′ between all SNPs [19]. Additional data from the International HapMap Genome Browser (http://www.hapmap.org) showed that this LD block does not extend beyond the *CUL*5 gene region (unpublished data).

There were 11 haplotypes observed in total in the three populations: six (1, 2, 3, 7, 8, and 10) haplotypes were shared among all racial groups, while five haplotypes were observed only in AA (Figure 1). In EA, six major haplotypes with frequencies between 0.07 and 0.43 represented 98.8% of all chromosomes. In AA, 11 haplotypes with frequencies between 0.03 and 0.21 represent 95.5% of all chromosomes, eight of which had a frequency below 10%. The haplotype distribution in HC closely resembled that of EA.

### *CUL5* Haplotypes Form Two Clusters Separated by Three Cluster-Tagging SNPs

To determine the evolutionarily relatedness of the haplotypes, an evolutionary tree of the *CUL5* haplotypes composed of 12 SNPs was reconstructed using the phylogenetic network method [20] (Figure 2C and 2D). The network comprised two main clusters (referred to here as cluster I and II), which differ at SNPs 3, 5, and 8 in the human. These are separated by the chimpanzee sequence representing the ancestral haplotype and the median vector representing possibly extant unsampled sequences or extinct ancestral sequences. Since the proxy SNPs 3, 5, and 8 separated and defined clusters I and II, we defined these as cluster-tagging (ct) SNPs (ctSNPs). Cluster I and II contain six and five haplotypes, respectively. All haplotypes in cluster I contain the ancestral alleles at SNP 3, 5, and 8 (G, A, and A, respectively), and all haplotypes in cluster II contain the derived allele at SNP 3, 5, and 8 (A, G, and G, respectively) (Figures 1, 2C, and 2D).

### Effect of *CUL5* SNPs and Haplotypes on Susceptibility to HIV-1 Infection

We compared the *CUL5* SNP allele and haplotype frequency distributions among highly exposed but uninfected individuals to HIV-1 seronegative individuals (SN) and HIV-1-infected seroconverter individuals (SC). No distortion of frequency distribution between risk groups was observed for any SNP or haplotype in AA or EA (unpublished data), suggesting that the *CUL5* genetic variation assessed herein has no obvious effect on susceptibility to HIV-1 infection.

### *CUL5* Clusters Were Associated with CD4^+^ T Cell Depletion in African Americans

**Figure 3:**
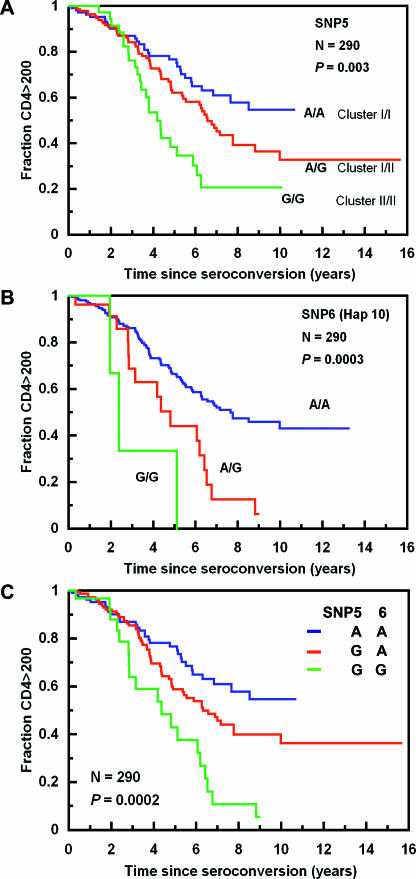
Kaplan-Meier Survival Analysis of *CUL5* Variants on Progression from Seroconversion to CD4^+^ T cells < 200 /mm^3^ in AA (A) Shows ctSNP5 (the cluster); (B) shows htSNP6 (Hap10); and (C) shows compound genotypes of ctSNP5 and htSNP6.

AA and EA were analyzed separately since the allele frequencies and haplotype structures differed between the two groups. To minimize the haplotypes and SNPs to be tested, we took advantage of the unique haplotype relationship in *CUL5* revealed in the haplotype network. We first tested the hypothesis that the two major clusters of haplotypes were differentially associated with disease progression in AA and EA. Cluster II identified by the ctSNP 5 G allele was significantly associated with accelerated rates of progression to CD4^+^ < 200 in both the unadjusted analysis and after adjusting (adj) for the confounding effects of *HLA* B57 and *HLA* homozygosity in the Cox proportional hazards model in AA (Table 1). When an additive genetic effect was tested in the Cox model analysis, the G allele significantly influenced the risk of dropping to CD4^+^ < 200 (relative hazards [RH]_adj_ = 1.47 and *p =* 0.009). No significant associations were found for AIDS-1987 (Table 1). Kaplan-Meier survival curves stratified for the ctSNP5 A/A, A/G, and G/G genotypes suggested an additive effect of the G allele (and by inference cluster II haplotypes) on progression to CD4^+^ < 200 (*p =* 0.003, log-rank) (Figure 3A). These results suggest that cluster II haplotypes, all of which carry ctSNP5 G, were associated with more rapid loss of CD4^+^ T cells, and that this effect is dose-dependent: individuals bearing one or two haplotypes from cluster II are at greater risk relative to individuals bearing any two haplotypes from cluster 1 (Figure 3A).

**Table 1:**
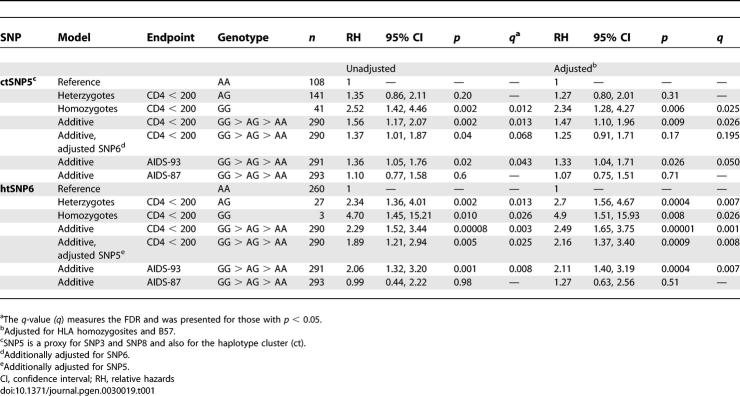
Effects of *CUL*5 SNPs on Progression to CD4^+^ T Cells < 200 and AIDS in African Americans

### *CUL5* Cluster I and II Contain Protective and Detrimental Haplotypes, Respectively

We next examined the role of each haplotype to identify specific high-risk haplotypes. The Cox model analysis indicated that the haplotype-tagging (ht)SNP6 G allele carried only on Hap10 ( *f* ≈ 5%) was a strong risk factor for CD4^+^ T cell depletion (RH_adj_ = 2.49 and *p =* 0.00001, for the additive model) (Figure 3B and Table 1). In a Kaplan-Meier survival plot, the htSNP6 G allele was a risk factor for CD4^+^ < 200: the three GG homozygous individuals developed CD4^+^ < 200 within five years (and all died within six years), and all A/G heterozgotes developed CD4^+^ < 200 within nine years of seroconversion (*p =* 0.0003, log-rank and *p =* 0.008, Wilcoxon) (Figure 3B).

Because Hap10 carries both the htSNP6 G allele and the ctSNP5 G allele, we tested ctSNP5 adjusting for the effects of htSNP6 within a Cox model. The ctSNP5 effect became nonsignificant (RH_adj_ = 1.25 and *p =* 0.170), suggesting that most of the association of ctSNP5 G allele and other cluster II haplotypes with CD4^+^ T cell loss was due to htSNP6. When considering ctSNP5 as a confounding covariate, the association remained robust for htSNP6 (RH_adj_ = 2.16 and *p =* 0.0009). However, not all of the ctSNP5 association with CD4^+^ T cell decline could be attributed to htSNP6. This can be readily observed in Figure 3C where individuals homozygous for cluster I haplotypes (ctSNP5 A/A) are protective relative to cluster II carriers, and the greatest risk is for carriers of the cluster II Hap10 (*p =* 0.0002, log-rank). This suggests that one or more ctSNP5 G-bearing haplotypes and the htSNP6 G-bearing Hap10 may be tracking the same functional allele elsewhere in the *CUL5* gene.

Hap1 and Hap6 in cluster I also were associated with delayed time to CD4^+^ < 200 (RH = 0.59, 0.41; *p* = 0.013, 0.02, respectively) (Table 2 and Table S2). None of the SNPs or haplotypes was significantly associated with progression to the AIDS late-stage endpoint AIDS-87 (Table 1 and Table S2).

**Table 2:**
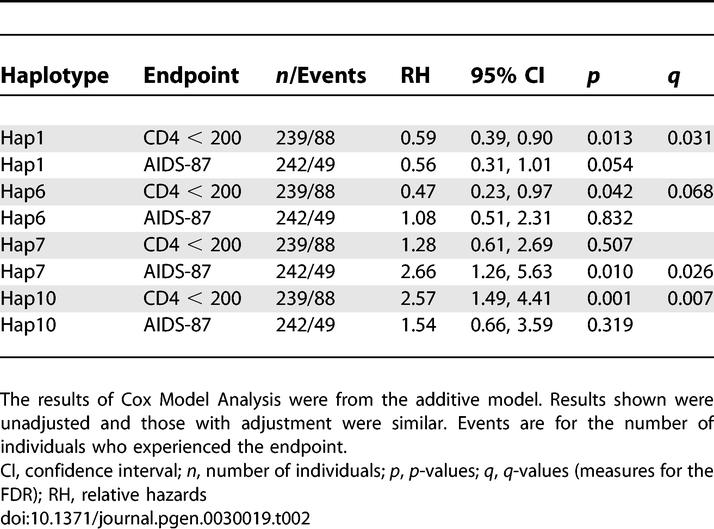
Effects of Selected *CUL5* Haplotypes on AIDS Progression in AA by Cox Model Analysis

### Relative Contribution of *CUL*5 Haplotypes on CD4^+^ T Cell Depletion

To evaluate the relative contributions of the protective Hap1 and Hap6 haplotypes and the accelerating Hap10 on time to CD4^+^ < 200, the Akaike Information Criteria (AIC) were used to select the best Cox proportional hazards model. The covariates *HLA* homozygosity and *HLA* B57 were used in the base mode (model 1). The smallest AIC was achieved for model 5 where all three haplotypes were added in model 5 (Table S3).

### Role of *CUL5* SNPs and Haplotypes on AIDS-Free Survival in European Americans

The six haplotypes in EA also form two clusters (Figure 2D). In the Cox model analysis, ctSNP5 showed no significant associations (Table S4), although weak associations were observed for two haplotypes and three SNPs. The cluster I haplotype Hap3 had an additive protective effect on rate of progression to CD4^+^ < 200 and AIDS 1993 (RH = 0.71–0.68 per allele, *p =* 0.026–0.006, respectively), and the cluster I Hap7 had a slight accelerating effect (CD4^+^ < 200, RH = 1.25, *p =* 0.056; AIDS-93, RH = 1.31, *p =* 0.010, respectively) (Table S2). Small effects were also observed for SNP4 (protective for AIDS-93, RH = 0.78, *p =* 0.03), SNP7 (risk for AIDS-93, RH = 1.24, *p =* 0.02), and SNP12 (risk for AIDS-93, RH = 1.2, *p =* 0.02) (Table S4).

### Effect of *CUL5* SNPs and Haplotypes on Longitudinal CD4^+^ T Cell Counts and HIV-1 RNA Levels in African Americans from the AIDS Link to the Intravenous Experience Cohort

CD4^+^ T cell counts and HIV-1 RNA levels were measured multiple times during the follow-up period for the AIDS Link to the Intravenous Experience (ALIVE) SC participants. An average of 7.8 and 6.8 measurements of CD4^+^ T cell counts and viral load, respectively, per patient for up to nine years from the seroconversion to 1997-censoring date were available for analysis. We evaluated the effects of ctSNP5 (representing all cluster II haplotypes) and htSNP6 (Hap10) on the longitudinal slope of CD4^+^ T cell count and HIV-1 RNA level over the clinical course using the linear mixed random effects model.

**Figure 4:**
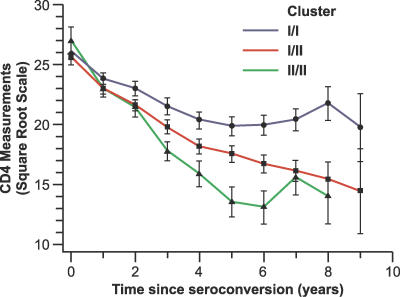
Arithmetic Mean of CD4^+^ T Cell Counts over Time in the ALIVE Cohort by ctSNP5 (the Cluster) The observation period was from seroconversion to the censoring date of July 31, 1997. CD4^+^ T cell counts were measured at 6-month intervals and were square-root transformed with standard error represented by the vertical bar.

The ctSNP5 G allele tends to be associated with gradient differences for CD4^+^ T cell slopes stratified by genotype (Figure 4 and Table 3): each copy of the ctSNP5 G allele was associated with −0.99 (*p=* 0.05) or −1.23 (*p =* 0.01) lower mean CD4^+^ T cell trajectory, over the observation periods from seroconversion to July 31, 1997 (date censored before highly active antiretroviral therapy [HAART]) or to July 31, 2004, respectively (Table 3). The htSNP6 G allele on Hap10 was strongly associated with a more rapid mean loss of CD4^+^ T cells: each copy of the ctSNP6 G allele was associated with −2.56 (*p =* 0.01) or −2.63 (*p =* 0.01) lower mean CD4^+^ T cell trajectory, for the observation periods from seroconversion to July 31, 1997 or to July 31, 2004, respectively (Table 3).

**Table 3:**
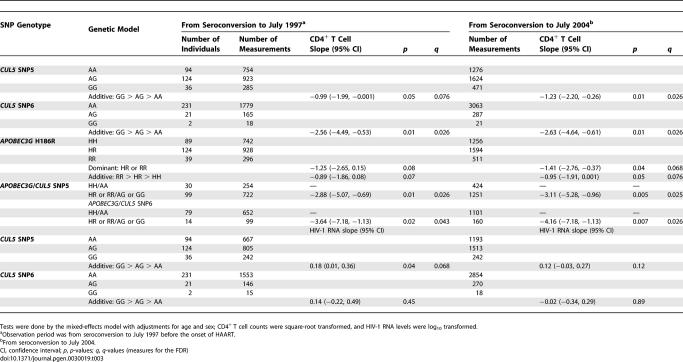
Effects of *CUL5* and *APOBEC3G* SNPs on the Longitudinal CD4^+^ T cell and HIV-1 RNA Slope Gradients in the ALIVE Cohort

For HIV-1 RNA level, a modest increase in HIV-1 RNA levels was observed for ctSNP5 under an additive model (+0.18, *p =* 0.04). No effect was detected for SNP6, possibly due to the low frequency of the minor allele and smaller number of HIV-1 RNA measurements available (Table 3).

### Interactions between *APOBEC3G*-H186R and *CUL5* SNPs

Because Cullin 5 affects Apobec3G's anti-HIV-1 activity, we reasoned that the *CUL5* cluster II haplotypes may interact with the *APOBEC3G*-186R allele, previously reported by us to be associated with accelerated CD4^+^ T cell depletion [18]. Using the group of individuals homozygous for both *APOBEC3G*-186H and cluster I haplotypes as a reference group, we tested the effects of different combinations of alleles on the mean CD4^+^ T cell trajectories using the mixed effects model (Table 3). We observed a significant additive interaction between *APOBEC3G*-186R carriers and ctSNP5 G or htSNP6 G carriers (−2.88, *p =* 0.01 and −3.64, *p =* 0.02, respectively), in the data censored in July 31, 1997. A stronger association (−3.11, *p =* 0.005 and −4.16, *p =* 0.007, respectively) was obtained using the extended followed-up time to July 31, 2004 (Table 3).

### Estimate of the False Discovery Rates

To account for the multiple comparisons made in this study, we estimated the *q*-value statistic to estimate the false discovery rate (FDR). The FDR was calculated incorporating all *p*-values from 122 tests performed for SNPs and haplotypes in the Cox model and mixed effects model. The *q*-value was obtained for each and all of the *p*-values, and the *q*-values for those with *p* ≤ 0.05 were presented. The associations for ctSNP5, htSNP6 with CD4^+^ < 200, and CD4^+^ T cell slope in AA as well as Hap1, Hap7 in AA, and Hap3 in EA had *q*-values below the stringent cutoff of 0.05 indicating a FDR of 5% for a given *p*-value (Tables 1–3 and Tables S2–S4).

### Electrophoretic Mobility-Shift Assay

**Figure 5:**
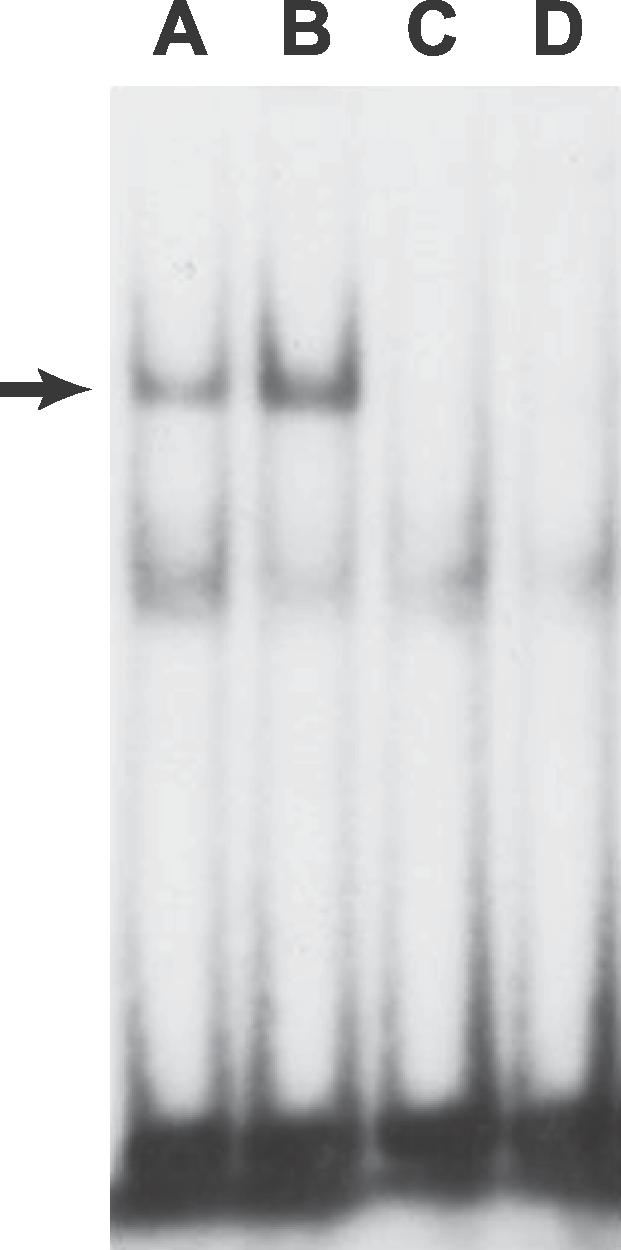
EMSA Analysis of *CUL5* SNP6 A/G Nuclear extracts from human T lymphocytes induced by IL-4 were bound to the oligonucleotide containing the SNP6 A allele (lane A) or G allele (lane B), without cold competitors. Lane C contained nuclear extracts, SNP6 A allele probe, and a 100-fold excess of its cold probe as competitor. Lane D contained nuclear extracts, SNP6 G allele probe, and a 100-fold excess of its cold probe as competitor. An arrow indicates the band showing differential binding of nuclear factor(s) to the oligonucleotides.

To explore the possibility that the SNP6 may differentially bind to nuclear proteins, probes containing the SNP6 A allele or the G allele were incubated with the nuclear extracts from human T lymphocytes stimulated with interleukin (IL)-4 in an electrophoretic mobility-shift assay (EMSA). A 1.85-fold or 1.75-fold increase in the band density was observed for the SNP6 G allele in comparison with the common A allele from two independent experiments, although no pattern changes were observed (Figure 5). A similar but weaker result was obtained in the IL-2 stimulated T cells (unpublished data). This suggests that the SNP6 G allele has higher binding affinity with unknown nuclear proteins.

## Discussion

The ability of Apobec3G to restrict HIV-1 replication through hypermutation is suppressed by HIV-1 Vif binding to *CUL5* gene product Cullin 5, leading to the polyubiquitination and degradation of Apobec3G and Apobec3F through the Cullin 5-Elongin B-Elongin C E3 ubiquitin ligase pathway [7,14,21]. To understand the role of genetic variation in the gene encoding Cullin 5 on HIV-1/AIDS, we examined 12 SNPs and their haplotypes on risk and progression of HIV-1 disease. The haplotypes formed two clusters defined by alleles at the proxy SNPs 3, 5, and 8. Relative to cluster I, cluster II haplotypes as a group were associated with faster CD4^+^ T cell decline as indicated by the Cox model analysis of survival to the endpoint of CD4^+^ < 200 and reaffirmed by the mixed effects model assessing CD4^+^ T cell trajectory over time. Individuals who were carriers of any two cluster II haplotypes progressed to CD4^+^ < 200 about 5.7 years faster than those who carried any two cluster I haplotypes. The effect of cluster II was largely attributable to Hap10, carrying both ctSNP5 G and htSNP6 G alleles. On the other hand, cluster I contains the two protective haplotypes, Hap1 and Hap6, which delayed CD4^+^ T cell loss. We also observed an additive interaction between *CUL5* cluster II haplotypes and *APOBEC3G*-H186R on CD4^+^ T cell slope, suggesting that Apobec3G and Cullin 5 likely confer independent effects on CD4^+^ T cell loss [18].

The genetic effects derived from multiple SNPs and haplotypes, and from the haplotype clusters, provide evidence that genetic variation in *CUL5* likely modifies the rate of disease progression of HIV-1 with the effects being stronger and more consistent among AA than EA. The differences observed between the two racial groups suggest that there are additional functional alleles in LD with the SNP markers assessed in this study.

To assess the possibility that the observed associations were due to the multiple tests, we estimated the FDR for all the tests performed. Although this test is quite conservative as there is considerable correlation among SNPs, haplotypes, and disease endpoints, the FDR for significant (*p* < 0.05) SNP and haplotype associations in both AA and EA was 5% or less. However, the gold standard for validation of genetic associations remains confirmation in other adequately powered studies.

Human Cullin 5 is a highly conserved 780-amino acid protein, differing by only seven amino acids from the rabbit VACM-1 protein [22,23]. The conservation of Cullin 5 amino acid sequence suggests that the Cullin 5 protein is under strong functional constraint and purifying selection. The fact that genetic effects observed for SNP5 and SNP6 were most robust under the additive genetic model suggests a dose-effect of the factors. It is thus most likely that the causal sequence or alleles lie in regulatory elements affecting *CUL*5 mRNA or protein levels. However, no promoter or nonsynonymous SNPs were reported among over 230 SNPs in the *CUL5* gene deposited in the dbSNP database as of July 6, 2006 and none were discovered when resequencing putative promoter and exonic regions in 188 individuals for this study.

In an in vitro electromobility gel shift assay, the DNA fragment carrying the G allele of SNP6, compared to the more common A allele, was found to show an increased binding affinity to nuclear proteins from human T lymphocytes. This suggests that SNP6 is a functional SNP that may affect the gene regulation or interaction. A higher level of Cullin 5 due to a potential up-regulation of *CUL5* gene would enhance the Cullin 5-Vif interaction, leading to stronger inhibition of antiviral protein Apobec3G and, hence, increased HIV-1 infectivity. In addition, a real-time PCR was performed to measure the mRNA expression levels of *CUL5* from EBV-transformed B cell lines in three, three, and six normal individuals carrying *CUL5* SNP5 genotypes AA, GG, and AG, respectively. No difference greater than 2-fold (minimal detection threshold) was detected between genotypes (unpublished data), though a more subtle difference could not be excluded. Little is known about *CUL5* regulation in different cell types or within CD4^+^ T cells infected by HIV-1. It is possible that SNPs in *CUL5* influence HIV-1 disease by alternative splicing, by affecting mRNA stability, or by unknown protein structural change, in addition to altered gene expression. The underlying mechanisms of *CUL5* genetic variation on HIV-1 warrant further investigation.

This study provides evidence that *CUL5* gene variation is associated with modified rates of CD4^+^ T cell loss. If confirmed, these findings demonstrate the epidemiologic importance of the interaction between HIV-1 Vif and human Cullin 5. The antiviral activity of both Apobec3G and Apobec3F have been shown to have antiviral activity that is fully suppressed by HIV-1 Vif through the Cullin 5 containing E3 ubiquitin ligase [7,14]. Depletion of Cullin 5 through RNA interference or overexpression of Cullin 5 mutants blocks the ability of HIV-1 Vif to suppress both Apobec3G and Apobec3F [14]. It is therefore possible that regulation of expression levels of *CUL5* genetic variants may affect the efficiency of Vif-mediated degradation of Apobec3F and Apobec3G. These in vitro studies, together with our findings that *CUL5* genetic factors modify the rate of CD4^+^ T cell loss, provide support for the development of inhibitors that block HIV-1 Vif and Cullin 5-Elongin B-Elongin C E3 ligase complex binding, aiming to limit or prevent degradation of these potent antiviral host factors.

## Materials and Methods

### Study participants.

Study participants were enrolled in five United States-based natural history HIV/AIDS cohorts. ALIVE is a community-based cohort of intravenous injection drug users in Baltimore enrolled in 1988–1989 [24], consisting of 92% AA. Multicenter AIDS Cohort Study (MACS) is a longitudinal prospective cohort of men who have sex with men from four U.S. cities: Chicago, Baltimore, Pittsburgh, and Los Angeles enrolled in 1984–1985 [25], consisting of 83% EA and 10% AA. The San Francisco City Clinic Study (SFCC) is a cohort of men who have sex with men originally enrolled in a hepatitis B study in 1978–1980 [26], consisting of 96% EA. Hemophilia Growth and Development Study (HGDS) is a multicenter prospective study that enrolled children with hemophilia who were exposed to HIV-1 through blood products between 1982 and 1983 [27], consisting of 72% EA and 11% AA. The Multicenter Hemophilia Cohort Study (MHCS) is a prospective study that enrolled persons with hemophilia [28], consisting of 90% EA and 6% AA. The individuals genotyped in this report consisted of HIV-1 SC, seroprevalents, SN, and high-risk exposed uninfected (HREU) for a total of 3,476 participants (2,169 EA and 1,307 AA). The numbers of EA and AA individuals studied in each disease category were as follows: SC = 659, 290; SN = 309, 336; HREU = 141, 82; respectively. Of 290 AA SC, 237, 42, five, and five were from ALIVE, MACS, MHCS, and HGDS, respectively.

The date of seroconversion after study enrollment was estimated as the midpoint between the last seronegative and first seropositive HIV-1 antibody test; only individuals with less than two years' elapsed time between the two tests were included in the seroconverter progression analysis. The censoring date was the earliest of the date of the last recorded visit, or December 31, 1995 for the MACS, MHCS, HGDS, and SFCC, or July 31, 1997 for the ALIVE cohort, to avoid potential confounding by HAART. The censoring date was extended in the ALIVE cohort because of delayed administration of HAART to this group [24,29]. The MACS, MHCS, SFCC, and ALIVE consists of both SC (infected after study enrollment) and seroprevalents (infected before study enrollment) individuals: because of the potential for frailty bias (missing the most rapid progressors to AIDS and death) among seroprevalents, only SC enrolled in the ALIVE, MACS, MHCS, and SFCC were used in the analysis. In addition, DNA samples from 110 normal blood donor HC were included to provide an estimate of allele frequencies in a major Asian population and to inform for future *CUL5* genetic studies in this population. This group was not used in the association analyses.

The study was approved by the Institutional Review Boards of participating institutes, and informed consent was obtained from the participants.

### Identification of SNPs.

A panel consisting of 94 DNA samples each from EA and AA was partially resequenced to discover novel *CUL5* polymorphisms. PCR primers were designed based on GenBank DNA sequence AP003307 and mRNA sequence NM_003478 to cover the putative promoter region, 5′ UTR, exons 15–19 encoding the C terminus that was shown to confer major function [7], and 3′ UTR of the *CUL5* gene. SNPs were obtained from dbSNP, HapMap (http://www.hapmap.org), and TaqMan SNP Genotyping Assay databases (http://myscience.appliedbiosystems.com). A total of 12 SNPs were selected for genotyping by considering SNP location, spacing, and at least 5% allele frequency (Figure 1A and 1B). Haplotype-tagging (ht)SNPs were given preference in the SNP selection.

### Genotyping of SNPs.

Genotyping was performed using TaqMan assays according to the manufacturer's manual (Applied Biosystems, http://www.appliedbiosystems.com). TaqMan primer and probes were designed by using the Primer Express software or by the Assay-by-Demand service of Applied Biosystems (Table S1). A total of eight water controls were included on each plate to monitor the potential PCR contamination, and 10% of SC and HREU samples were genotyped twice. The genotypes obtained were free of water contamination or of inconsistencies between duplicates. SNP5 was genotyped twice using two different sets of primers and probes, and the results were identical.

### Statistical analysis.

Analyses were conducted using the statistical packages SAS version 9.0 (SAS Institute, http://www.sas.com). Conformity to the genotype frequencies expected under Hardy-Weinberg equilibrium was examined for each SNP. The genetic effects of SNPs on HIV-1 infection susceptibility were assessed by comparing allelic frequencies between HIV-1 HREU and HIV-1 SC participants using the Fisher's exact test. Kaplan-Meier survival statistics and the Cox proportional hazards model were used to assess the effects of SNPs and haplotypes on the rate of progression to AIDS. We considered three separate endpoints reflecting advancing AIDS pathogenesis for SC: (1) HIV-1 infection plus a decline of CD4^+^ T cell counts < 200 cells/mm^3^ (CD4^+^ < 200); (2) the 1993 Centers for Disease Control and Prevention (CDC) definition of AIDS (AIDS-93): HIV-1 infection plus a CD4^+^ T cell count of <200/mm^3^ or AIDS-defining conditions [30]; (3) the 1987 CDC definition of AIDS (AIDS-87): HIV-1 infection plus AIDS-defining illness [31]. The significance of genotypic associations and RH was determined by unadjusted and adjusted Cox model regression analyses. For each SNP, we compared the minor allele genotypes to the most common genotype as a reference group. To determine if there was an additive effect of SNPs or alleles, the additive genetic model was tested by comparing survival in persons carrying two to one minor alleles to the homozygous reference group, coded as 2, 1, and 0, respectively, in the regression analysis: RH reflects effects of each copy of the allele or the haplotype. All *p-*values were 2-tailed. Genetic factors previously shown to affect progression to AIDS in EA and AA groups were included as confounding covariates in the adjusted Cox model analysis: *CCR5* Δ32 [32]; *CCR2*-64I [33]; *CCR5*-P1 [34]; *HLA*-B*27 [35]; *HLA*-B*57 [35]; *HLA*-B*35Px group (including HLA-B*3502, B*3503, B*3504, and B*5301) [36]; and *HLA* class I homozygosity [37] for EA; *HLA*-B*57 and *HLA* class I homozygosity for AA. *CCR2*-64I, *HLA*-B*27, and *HLA*-B*35Px were not considered as covariates in AA due to absent or weak effects in our AA participants, and *CCR5* Δ32 was not considered due to its rarity in AA. Participants were stratified by sex and by age at seroconversion: 0–20, >20–40, and >40 years [38].

To evaluate which haplotype or combination of haplotypes predicted survival, the AIC generated in the Cox model was used as the model selection criterion [39]. Among models tested, the one with the smallest value of AIC was considered to be the best model.

To account for the multiple comparison tests performed in our analysis, we estimated the *q*-value measuring FDR, which is defined as the proportion of statistical tests called significant that are actually false-positive (http://faculty.washington.edu/jstorey/qvalue)[40]. The approach of computing *q*-values, also known as FDR adjusted *p*-values, was considered more powerful than the Bonferroni procedure when there is high degree of LD among the markers [41]. FDR is a recommended procedure to correct for multiple comparisons in genetic association studies [42].

### *CUL5* genotype, HIV-1 RNA, and CD4^+^ T cells.

The repeated measurements of HIV-1 RNA levels and CD4^+^ T cell counts over time were modeled using the random effects linear models from HIV-1 SCs enrolled in the ALIVE study, adjusted for sex and age [18]. This model provides estimates of mean CD4^+^ T cell measurements over time while accounting for the correlation of repeated measurements within each individual. In all models absolute CD4^+^ T cell counts were square-root transformed, and plasma HIV-1RNA levels were log_10_ transformed to better comply with model assumptions. A likelihood ratio test was used as a test of significance. Two observation periods were used: (1) from seroconversion to the censoring date of July 31, 1997, before any impact of HAART was observed in the ALIVE cohort [24]; and (2) to increase the number of observations, from seroconversion to July 31, 2004, the last date for which CD4^+^ T cell and HIV-1 RNA levels were available for analysis.

### LD and haplotype structure.

Pairwise LD was quantified using the absolute value of Lewontin's D′ [43]. Absolute values of D′ range from 0 for independence to 1 for complete LD between the pairs of loci. LD plots were generated utilizing Haploview version 3.11 (https://www.broad.mit.edu/mpg/haploview) [44]. A triangular matrix of D′ value was used to demonstrate LD patterns within AA and EA. Haplotype blocks were defined with a default algorithm based on confidence intervals of D′ [19]. Haplotypes were inferred by the expectation maximization algorithm [45].

To depict the relationships among inferred haplotypes and evolutionary history of the genetic variants at the *CUL5* locus, a minimum mutation phylogenetic network was constructed by using NETWORK package, based on the Reduced Median (RM) network method (http://fluxus-technology.com/sharenet.htm) [20]. A chimpanzee sequence was included as an out-group and was aligned to the paralogous human sequence to infer the derived or ancestral state for each human SNP.

### EMSA.

Cell culture and EMSA were performed as described [46,47]. Freshly explanted human T lymphocytes were obtained from normal donors, purified by isocentrifugation, and activated for 72 h with 1 mg/ml phytohemaglutinin (PHA) in RPMI 1,640 medium containing 10% fetal calf serum (FCS) (Sigma, http://www.sigmaaldrich.com), 2 mM L-glutamine, and penicillin-streptomycin (50 IU/ml and 50 mg/ml, respectively). T lymphocytes were made quiescent by washing and incubating for 24 h in RPMI 1,640 medium containing 1% FCS before exposure to cytokines. Cells were then stimulated with 100 nmol/l IL-4 (PeproTech, http://www.peprotech.com) or 100 nmol/l human rIL-2 (Hoffmann-La Roche, http://www.rocheusa.com) at 37 °C for 10 min. Cell pellets were frozen at −70 °C. The probe sequences were 5′-CAGTTGAACATaCCTTGTTAGGA-3′ for SNP6 A allele and 5′-CAGTTGAACATgCCTTGTTAGGA-3′ for SNP6 G allele. In cold oligonucleotide competition assay, 100-fold excess of unlabeled probe was added as a competitor. The band density was measured by the software ImageJ (http://rsb.info.nih.gov/ij/index.html).

## Supporting Information

Table S1 Genotyping Methods for *CUL5* SNPs (18 KB XLS)

Table S2 Cox Model Analysis of *CUL5* Haplotypes (22 KB XLS)

Table S3 Selection of the Top Models for *CUL5* Haplotypes Using AIC (15 KB XLS)

Table S4 Cox Model Analysis of *CUL5* SNPs in EA (17 KB XLS)

### Accession Numbers

The Entrez Gene databank (http://www.ncbi.nlm.nih.gov/entrez/query.fcgi?db=gene) accession numbers for the genes discussed in this paper are *Cullin 5* (8065), *Elongin B* (6923), *Elongin C* (6921), *Rbx1* (9978), *Apobec3G* (60489), *Apobec3F* (200316), and *HIV-1 Vif* (155459).

The GenBank (http://www.ncbi.nlm.nih.gov) accession number for chimpanzee *CUL5* sequence is NW_113990.

## Abbreviations

AA: African Americans
adj: adjusting
AIC: Akaike Information Criteria
ALIVE: AIDS Link to the Intravenous Experience
Apobec3: apolipoprotein B mRNA editing enzyme, catalytic polypeptide-like 3
ct: cluster tagging
EA: European Americans
EMSA: electrophoretic mobility-shift assay
FDR: false discovery rate
HC: Han Chinese
HIV-1: HIV type 1
ht: haplotype tagging
IL: interleukin
LD: linkage disequilibrium
MACS: Multicenter AIDS Cohort Study
MHCS: Multicenter Hemophilia Cohort Study
RH: relative hazards
SC: seroconverters
SN: seronegatives
SNP: single nucleotide polymorphism
Vif: viral infectivity factor
